# 172. Impact of COVID-19 Pandemic on Healthcare-associated Infections (HAIs) in a Large Network of Hospitals

**DOI:** 10.1093/ofid/ofab466.172

**Published:** 2021-12-04

**Authors:** Sonali D Advani, Sonali D Advani, Emily Sickbert-Bennett, Elizabeth Dodds Ashley, Andrea Cromer, Yuliya Lokhnygina, Alicia Nelson, Ibukunoluwa Akinboyo, Lauren DiBiase, David J Weber, Deverick J Anderson

**Affiliations:** 1 Duke University School of Medicine, Duke Infection Control Outreach Network, Durham, NC; 2 UNC Health Care, Chapel Hill, NC; 3 Duke Center for Antimicrobial Stewardship and Infection Prevention, Durham, NC; 4 Duke Infection Control Outreach Network (DICON), Inman, South Carolina; 5 Duke University School of Medicine, Durham, North Carolina; 6 Duke University, Durham, NC; 7 University of North Carolina School of Medicine, Chapel Hill, NC

## Abstract

**Background:**

The COVID-19 pandemic had a considerable impact on US healthcare systems, straining hospital resources, staff, and operations. Our objective was to evaluate the impact of COVID-19 pandemic on incidence and trends of healthcare-associated infections (HAIs) in a network of hospitals.

**Methods:**

This was a retrospective review of central-line-associated bloodstream infections (CLABSIs), catheter-associated urinary tract infections (CAUTIs), C. difficile infections (CDI), and ventilator-associated events (VAE) in 51 hospitals from 2018 to 2021. Descriptive statistics were reported as mean hospital-level monthly incidence rates (IR) and compared using Poisson regression GEE models with period as the only covariate. Segmented regression (SR) analysis was performed to estimate changes in monthly IR of CAUTIs, CLABSIs and CDI in the baseline period (01/2018 – 02/2020) and the Pandemic period (03/2020 – 03/2021). SR model was not appropriate for VAE based on the plot. All models were constructed using SAS v.9.4 (SAS Institute, Cary NC).

**Results:**

Compared to the baseline period, CLABSIs increased significantly by 50% from 0.6 to 0.9/ 1000 catheter days (P< 0. 001). In contrast, no significant changes were identified for CAUTI (P=0.87). Similar trends were seen in SR models for CLABSI and CAUTI (Figures 1, 2 and Table 1). While overall CDIs decreased significantly from 3.5 to 2.5/10,000 patient days in the pandemic period (P< 0.001), SR model showed increasing pandemic trend change (Figure 3). VAEs increased > 700% from 6.9 to 59.7/1000 ventilator days (P=0.15), but displayed considerable variation during the pandemic period (Figure 4). Compared to baseline period, there was a significant increase in central line days (647 vs 677, P=0.02), ventilator days (156 vs 215, P< 0.001), but no change in urinary catheter days (675 vs 686, P=0.32) during the pandemic period.

Figure 1: Segmented Regression model showing baseline and pandemic period trends of CLABSI

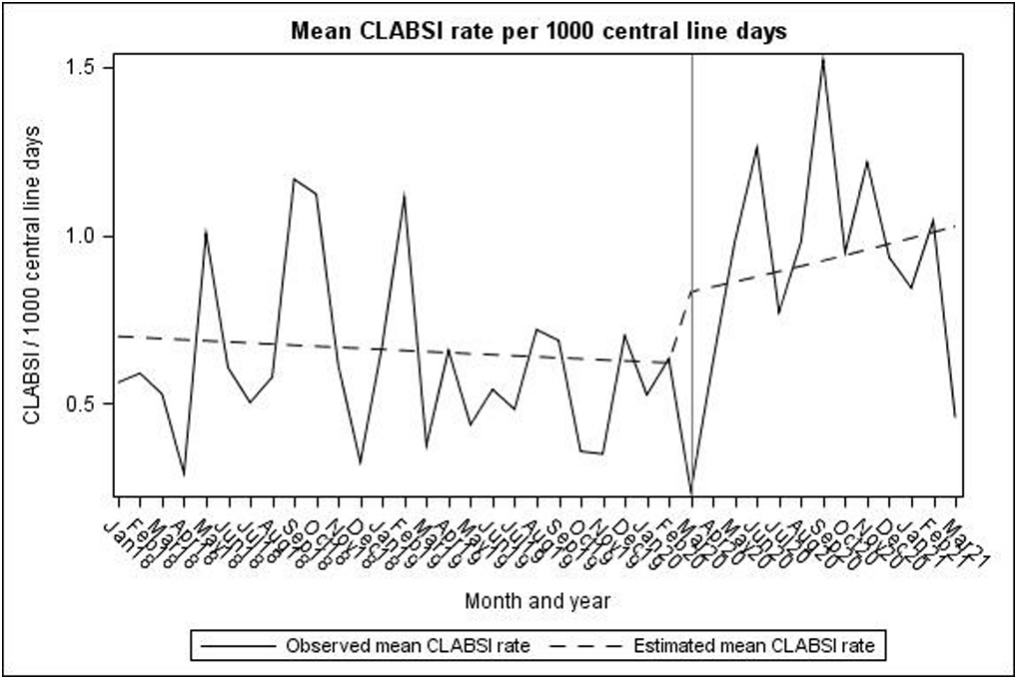

Figure 2: Segmented Regression model showing baseline and pandemic period trends of CAUTI

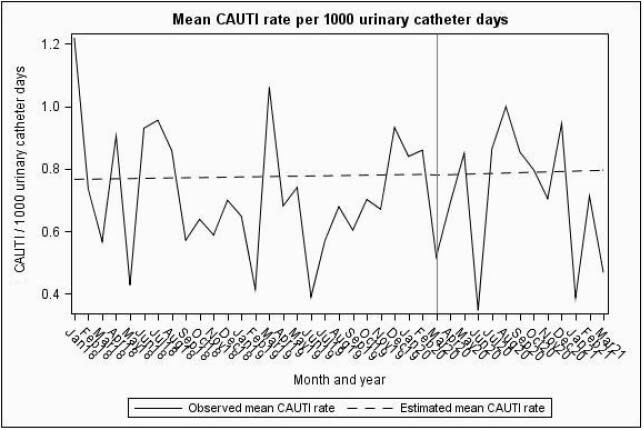

Figure 3: Segmented Regression model showing baseline and pandemic period trends of C. difficile (HO-CDI) infections

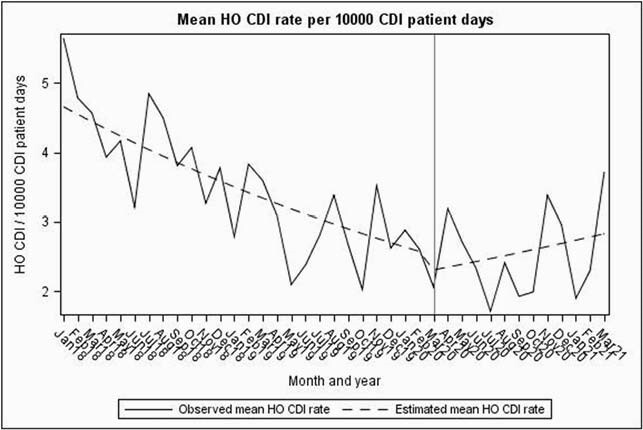

**Conclusion:**

The COVID-19 pandemic was associated with substantial increases in CLABSIs and VAEs, no change in CAUTIs, and an increasing trend in CDI incidence. These variations in trends of different HAIs are likely due, in part, to unique characteristics of the underlying infection, resource shortages, staffing concerns, increased device use, changes in testing practices, and the limitations of surveillance definitions.

Figure 4: Trend of Ventilator-Associated Events (VAE) in the baseline and pandemic period (Segmented Regression model not appropriate)

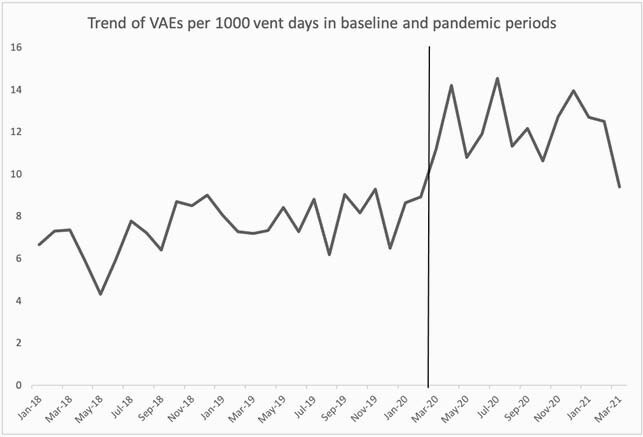

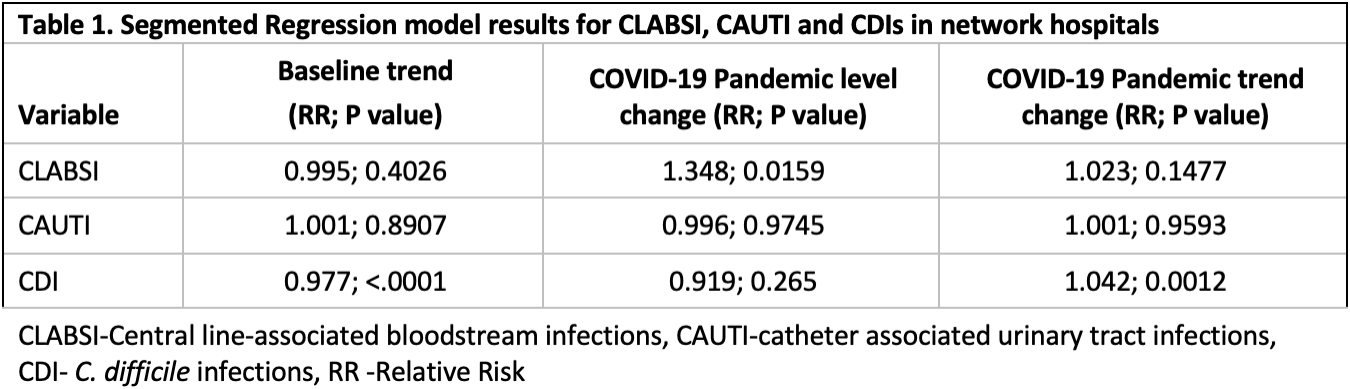

**Disclosures:**

**Sonali D. Advani, MBBS, MPH**, Nothing to disclose **David J. Weber, MD, MPH**, Merck (Individual(s) Involved: Self): Consultant; PDI (Individual(s) Involved: Self): Consultant; Pfizer (Individual(s) Involved: Self): Consultant; Sanofi (Individual(s) Involved: Self): Consultant; UVinnovators (Individual(s) Involved: Self): Consultant

